# Gold-Nanoparticle-Deposited TiO_2_ Nanorod/Poly(Vinylidene Fluoride) Composites with Enhanced Dielectric Performance

**DOI:** 10.3390/polym13132064

**Published:** 2021-06-23

**Authors:** Pornsawan Kum-onsa, Narong Chanlek, Jedsada Manyam, Prasit Thongbai, Viyada Harnchana, Nutthakritta Phromviyo, Prinya Chindaprasirt

**Affiliations:** 1 Materials Science and Nanotechnology Program, Faculty of Science, Khon Kaen University, Khon Kaen 40002, Thailand; pornsawan.kumonsa@gmail.com; 2 Synchrotron Light Research Institute (Public Organization), 111 University Avenue, Muang District, Nakhon Ratchasima 30000, Thailand; Narong@slri.or.th; 3 Nanotechnology Center (NANOTEC), National Science and Technology Development Agency (NSTDA), Pathum Thani 12120, Thailand; jedsada@nanotec.or.th; 4 Department of Physics, Faculty of Science, Khon Kaen University, Khon Kaen 40002, Thailand; viyada@kku.ac.th; 5 Institute of Nanomaterials Research and Innovation for Energy (IN-RIE), NANOTEC-KKU RNN on Nanomaterials Research and Innovation for Energy, Khon Kaen University, Khon Kaen 40002, Thailand; 6 Sustainable Infrastructure Research and Development Center, Department of Civil Engineering, Faculty of Engineering, Khon Kaen University, Khon Kaen 40002, Thailand; nutthaphrom@gmail.com (N.P.); prinya@kku.ac.th (P.C.)

**Keywords:** gold nanoparticle, titanium dioxide nanorod, poly(vinylidene fluoride), heat treatment, hybrid nanoparticle, modified Turkevich method

## Abstract

Flexible dielectric polymer composites have been of great interest as embedded capacitor materials in the electronic industry. However, a polymer composite has a low relative dielectric permittivity (ε′ < 100), while its dielectric loss tangent is generally large (tanδ > 0.1). In this study, we fabricate a novel, high-permittivity polymer nanocomposite system with a low tanδ. The nanocomposite system comprises poly(vinylidene fluoride) (PVDF) co-filled with Au nanoparticles and semiconducting TiO_2_ nanorods (TNRs) that contain Ti^3+^ ions. To homogeneously disperse the conductive Au phase, the TNR surface was decorated with Au-NPs ~10–20 nm in size (Au-TNRs) using a modified Turkevich method. The polar β-PVDF phase was enhanced by the incorporation of the Au nanoparticles, partially contributing to the enhanced ε′ value. The introduction of the Au-TNRs in the PVDF matrix provided three-phase Au-TNR/PVDF nanocomposites with excellent dielectric properties (i.e., high ε′ ≈ 157 and low tanδ ≈ 0.05 at 1.8 vol% of Au and 47.4 vol% of TNRs). The ε′ of the three-phase Au-TNR/PVDF composite is ~2.4-times higher than that of the two-phase TNR/PVDF composite, clearly highlighting the primary contribution of the Au nanoparticles at similar filler loadings. The volume fraction dependence of ε′ is in close agreement with the effective medium percolation theory model. The significant enhancement in ε′ was primarily caused by interfacial polarization at the PVDF–conducting Au nanoparticle and PVDF–semiconducting TNR interfaces, as well as by the induced β-PVDF phase. A low tanδ was achieved due to the inhibited conducting pathway formed by direct Au nanoparticle contact.

## 1. Introduction

With recent developments in the electronic industry, dielectric polymer composite materials have attracted increasing interest for a wide range of applications, such as energy storage devices, dielectric capacitors, and electromechanical actuators [1,2]. Poly(vinylidene fluoride) (PVDF) has been used as a dielectric polymer material due to its high energy density, high electric break down field, and flexibility [3,4]. However, the relative dielectric permittivity (ε′) of PVDF is too low (≈10 [3]) for electronic applications.

Many studies have attempted to fabricate polymer composites with high ε′ values by incorporating fillers into the PVDF matrix. Two-phase ceramic/polymer and metal/polymer composites have been synthesized and widely studied for improving the dielectric performance of polymer composite materials [5,6,7,8,9,10,11]. Several ceramic/polymer composites, such as CaCu_3_Ti_4_O_12_/PVDF [5,12], CaCu_3_Ti_4_O_12_/polystyrene [13], BaTiO_3_/PVDF [6], Ba_0.5_Sr_0.5_TiO_3_/P(VDF-CTFE) [14], and Ba_0.6_Sr_0.4_TiO_3_/PVDF [15], have high ε′ values (~50–80 at 1 kHz). The ε′ of a ceramic/polymer composite is generally below 100 even at a high ceramic loading (50 vol%), while its dielectric loss tangent (tanδ) is also elevated (>0.1 at 1 kHz and ~25 °C) [5,16]. However, metal/polymer composites, such as Ni/PVDF, Ni/P(VDF-CTFE) [17,18], MWCNT/PVDF [8,19], and Ag/PVDF [7,20], can exhibit significantly higher ε′ at low concentrations of conducting fillers than ceramic/PVDF composites. It is difficult to maintain the filler loading at the percolation threshold (*f*_c_) to achieve a high relative permittivity. Metal/polymer composites generally exhibit significantly large tanδ and electrical conductivity (σ) values at *f*_c_. It should be noted that the metal/polymer composites with extreme ε′ values also have high tanδ and σ, which limits the practical applications of these metal/polymer composites.

Owing to such challenges, developing polymer composites with high ε′ and low tanδ values is desirable. Several researchers have studied and reported three-component composites comprising metal, ceramic, and polymer matrices, such as Ba(Fe_0.5_Nb_0.5_)O_3_/Ni/PVDF, Ni/CaCu_3_Ti_4_O_12_/PVDF, Ni/BaTiO_3_/PVDF, Na_0.5_Bi_0.5_Cu_3_Ti_4_O_12_/MWCNTs/PVDF, and Ag/Na_0.5_Bi_0.5_Cu_3_Ti_4_O_12_ [21,22,23,24,25]. In particular, a novel composite with structured hybrid fillers has been of great interest. Recently, many studies on PVDF-based composites filled with hybrid nanoparticles have been reported. Luo et al. [26] reported a novel polymer composite filled with Ag-BaTiO_3_ hybrid nanoparticles. This Ag-BaTiO_3_/PVDF composite exhibited a high ε′ (160) with tanδ ≈ 0.11 at a filler volume fraction (*f*_Ag-BT_) of 0.568. This tanδ value is much lower than those reported in many conventional three-phase polymer composites; unfortunately, it is still much larger than 0.05, which is an acceptable value for capacitor applications. Although incorporating Ag-BaTiO_3_ hybrid nanoparticles can increase the ε′ of a composite, the ε′ of ferroelectric BaTiO_3_ is generally strongly dependent on its Curie temperature. Furthermore, most ferroelectric oxides are piezoelectric, which results in mechanical resonance in the device during charging and discharging, thereby limiting its reliability [27].

Rutile-TiO_2_ is one of the most widely used oxides in electronic materials, sensors, and semiconductors [28,29]. Furthermore, rutile-TiO_2_ can exhibit colossal dielectric properties when a minor portion of Ti^4+^ is reduced to Ti^3+^ due to the existence of oxygen vacancies and/or substitution by pentavalent ions (e.g., Nb^5+^ or Ta^5+^). Polaron-like electron hopping between Ti^3+^ and Ti^4+^ ions can cause a significant increase (by a factor of ~10^4^) in dielectric permittivity [30]. Since TiO_2_ is not a ferroelectric ceramic, TiO_2_ nanoparticles were used as a filler in various polymer composites [31,32,33]. Unfortunately, the ε′ values of the TiO_2_/polymer composites are still significantly low owing to the low ε’ of the TiO_2_ nanoparticles. Polymer composites filled with modified TiO_2_ nanoparticles such as Ag-TiO_2_ hybrid particles and Ag@TiO_2_ core–shell structures were developed to enhance ε′ [34,35,36,37]. Although these composites can exhibit high ε′ values of ~60–150, large tanδ values are generally obtained (~0.1–1) at high filler concentrations (70 vol%) [34,35]. Among various metal nanoparticles, gold nanoparticles are widely used as fillers to improve the insulation properties of polymer materials because they are nontoxic and less likely to be oxidized [38]. A significantly enhanced ε′ (~54‒118) and low tanδ (<0.06) were achieved in Au-BaTiO_3_/PVDF [39] and Au-BiFeO_3_/PVDF, with only a small amount of Au in the third phase of each polymer composite (*f*_Au_ < 0.02) [40]. According to previous works [39,40], the Au-BaTiO_3_/PVDF and Au-BiFeO_3_/PVDF composites not only exhibited high ε′ values, but their tanδ and σ were also suppressed due to the incorporation of the Au nanoparticles. Therefore, the conductive Au phase nanoparticle is one of the most interesting conductive phases for use as a filler in three-phase polymer composites.

To the best of our knowledge, there is a lack of substantial information on polymer composites incorporated with Au-TiO_2_ hybrid nanoparticles. Therefore, in this study we aimed to fabricate a novel nanocomposite comprising a PVDF polymer matrix, Au nanoparticles, and TiO_2_ nanorods (TNRs). TNRs have higher surface areas than spherical TiO_2_; therefore, they lead to stronger interfacial polarization and a significantly enhanced ε′. Herein, Au-TNR/PVDF nanocomposites with enhanced ε′ and low tanδ were fabricated. A modified Turkevich method was used to attach Au onto the surfaces of the TNRs. The Au-TNR/PVDF nanocomposites were prepared through liquid-phase-assisted dispersion and hot-pressing methods. Several properties of these nanocomposites such as their morphologies, microstructures, phase structures, chemical stages, and dielectric properties were investigated, and the significantly improved dielectric properties of the nanocomposites are discussed.

## 2. Experimental Section

### 2.1. Preparation of Heat-Treated TNRs

TNRs (99.5% purity) with particle size <100 nm were purchased from Sigma-Aldrich. Heat treatment at 500 °C for 3 h in air was performed on the TNRs to evaporate the moisture.

### 2.2. Preparation of Au-TNR Hybrid Nanoparticles

Au-TNR hybrid nanoparticles were prepared through a modified Turkevich method. The corresponding procedure is described as follows: heat treatment of TNR powder was carried out by ultrasonically dispersing the powder in deionized water for 30 min. Then, the white TNR suspension was stirred using a magnetic stirrer at ~25 °C for 30 min, after which 1 mM HAuCl_4_·3H_2_O was dissolved in the TNR solution under constant stirring. After the solution was heated to 300 °C, 38.8 mM of sodium citrate (>99.0%, Sigma-Aldrich) solution was dissolved in the TNR solution. To ensure a complete reaction, the suspension was stirred until its color changed from white to purple. The purple suspension was sequentially cooled to room temperature, centrifuged at 8500 rpm, and washed several times with deionized water. Finally, Au-TNR hybrid nanoparticles were obtained without agglomeration by freeze-drying.

### 2.3. Preparation of Au-TNR/PVDF Nanocomposites

Au-TNR/PVDF nanocomposites containing Au-TNR fillers with different *f*_Au_ and *f*_TNRs_ values were prepared through liquid-phase-assisted dispersion and hot-pressing methods. First, Au-TNR hybrid nanoparticles and the PVDF powder (M_w_ ~ 534,000, Sigma-Aldrich) were mixed by ball-milling with ZrO_2_ in ethanol for 3 h. Second, the mixture was dried at 80 °C to evaporate ethanol, after which the mixed powder was pressed at 200 °C for 30 min at 10 MPa. Finally, the Au-TNR/PVDF nanocomposite sample, with a diameter and thickness of ~12 mm and ~0.5–1 mm, respectively, was obtained at room temperature. Au-TNR/PVDF nanocomposite samples with *f*_Au-TNRs_ = 0.094, 0.216, 0.294, 0.383, 0.492, and 0.624 are referred to as Au-TNRs/PVDF-1, Au-TNRs/PVDF-2, Au-TNRs/PVDF-3, Au-TNRs/PVDF-4, Au-TNRs/PVDF-5, and Au-TNRs/PVDF-6, respectively. The separated volume fractions of Au and TNRs for each composite sample are listed in Table 1.

### 2.4. Characterization

The phase structures of the PVDF filler and Au-TNR/PVDF nanocomposites were characterized by X-ray diffractometry (XRD, PANalytical, EMPYREAN). The surface morphologies of Au, TNRs, and the Au-TNR nanoparticles were revealed using transmission electron microscopy (TEM, FEI Tecnai G2 20). The chemical composition of each element in the Au-TNR hybrid nanoparticles was analyzed by X-ray photoelectron spectroscopy (XPS, PHI5000 VersaProbe II, ULVAC-PHI, Japan) at the SUT-NANOTEC-SLRI Joint Research Facility, Synchrotron Light Research Institute (SLRI), Thailand. The fracture microstructures, distributions, and percentages of each element in the Au-TNR/PVDF nanocomposites were investigated by focused ion beam–field emission scanning electron microscopy (FIB–FESEM, FEI Helios Nanolab G3 CX). The samples were fractured using liquid N_2_ and their surfaces were sputtered with Au before SEM characterization. The crystalline phases of the nanocomposites were determined using Fourier-transform infrared spectroscopy (FTIR, Bruker, TENSOR27) in the 700–1800 cm^−1^ wavelength range. The dielectric properties of the samples were analyzed using an impedance analyzer (KEYSIGHT E4990A) in the 10^2^–10^6^ Hz and −60–150 °C frequency and temperature ranges, respectively, with an oscillation voltage of 0.5 V. Before any dielectric measurement, both sides of each circular sample were coated with Ag to form electrodes.

## 3. Results and Discussion

**Figure 1:**
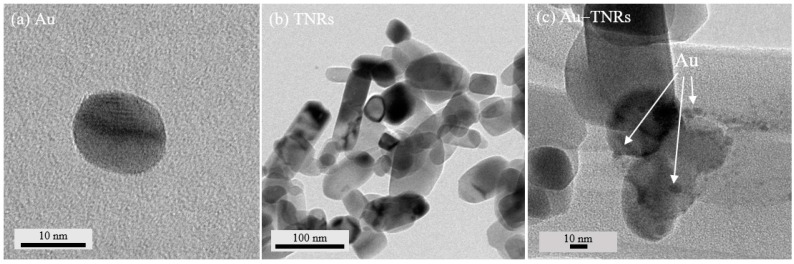
TEM images of (**a**) Au, (**b**) TNRs, and (**c**) Au-TNR hybrid nanoparticles.

Figure 1 displays TEM images showing the morphologies of the Au, TNRs, and Au-TNR hybrid nanoparticles. The Au nanoparticles are spherical with diameters of 10–20 nm. Meanwhile, the heat-treated TNRs are rod-shaped with slightly different aspect ratios, while some Au clusters are dotted on the TNR surfaces of the Au-TNR hybrid nanoparticles, revealing that the Au nanoparticles successfully formed on the TNR surfaces.

**Figure 2:**
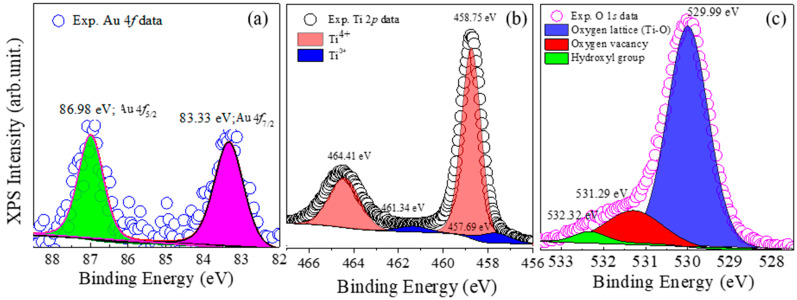
XPS spectra of Au-TNR hybrid nanoparticles; (**a**) Au 4*f*, (**b**) Ti 2*p*, and (**c**) O 1*s*.

Figure 2 shows XPS spectra of the Au-TNR powder. As shown in Figure 2a, Au 4*f* peaks were observed at 83.33 and 86.98 eV, which are assigned to Au 4*f*_7/2_ and Au 4*f*_5/2_, respectively [41,42]. This confirmed the existence of Au in the prepared Au-TNR powder. As shown in Figure 2b, small Ti 2*p* peaks were observed at binding energies of 457.69 and 461.34 eV, respectively, corresponding to the presence of Ti^3+^. Ti 2*p* signals was observed at binding energies of 458.75 and 464.41 eV, indicating the presence of Ti^4+^ [43]. The Ti^3+^/Ti^4+^ ratio was found to be 7.52%. Figure 2c shows three of O 1*s* XPS peaks; the peak at 529.99 eV can be attributed to the oxygen lattice (Ti–O) [28,43]. Additional peaks were observed at 531.29 and 532.32 eV, which can be attributed to the oxygen vacancy in the rutile structure [28] and hydroxyl groups [43], respectively. The detected Ti^3+^ in the Au-TNR powder is likely to have originated from oxygen vacancies, which can be explained by Equations (1) and (2).

(1) $$O_{O}^{x}\to \frac{1}{2}O_{2}+V_{O}^{••}+2e^{-}$$

(2) $$Ti^{4+}+e^{-}\to Ti^{3+}$$

The presence of the Ti^3+^ ions can cause a significant increase in conductivity, thereby leading to electron hopping between the Ti^3+^ and Ti^4+^ ions under an applied electric field. The XPS results confirmed the existence of Au, Ti^3+^, and oxygen vacancies, which affected ε′ enhancement in the Au-TNR/PVDF nanocomposites.

**Figure 3:**
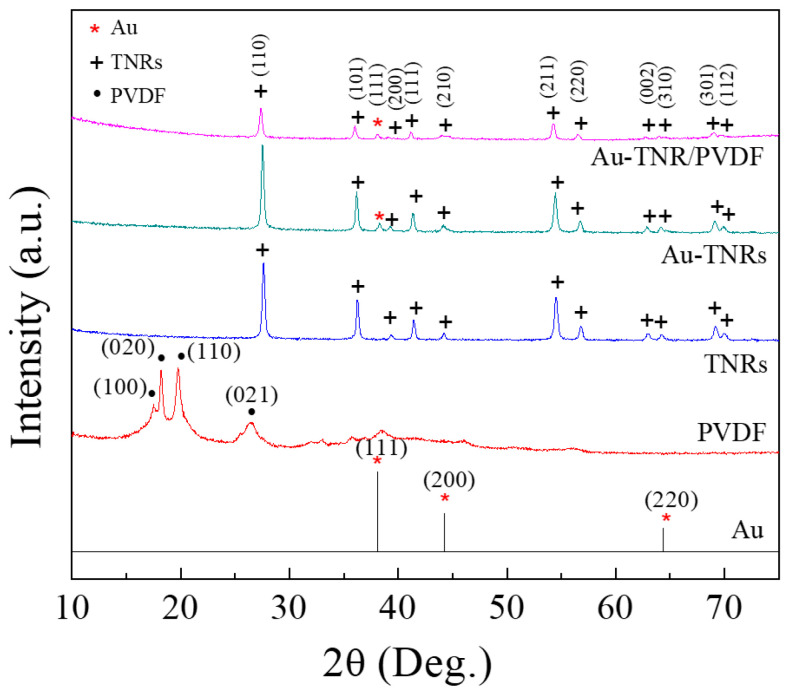
XRD patterns of the Au standard data Au standard (JCPDS 00-00-1172), TNRs, fabricated Au-TNR hybrid nanoparticles, and Au-TNR/PVDF-4 nanocomposite.

The XRD patterns of Au, PVDF, TNRs, Au-TNR nanoparticles, and Au-TNR/PVDF nanocomposites were obtained in the 10–80° 2θ range, as shown in Figure 3. The XRD pattern of the PVDF polymer corresponds to the (100), (020), (110), and (021) planes of the α-phase [4]. The XRD pattern of the TNRs showed peaks similar to those of the tetragonal structure of the rutile phase according to the standard reported in JCPDS 21-1276; no impurity phase was detected. In the case of the Au-TNR hybrid nanoparticles and Au-TNR/PVDF nanocomposites, the XRD peak for Au can be observed at 2θ ≈ 38.11 and assigned as a (111) plane (JCPDS 00-00-1172), confirming the existence of Au in the hybrid particles and Au-TNR/PVDF nanocomposites. Therefore, the Au nanoparticles were confirmed to exist in the Au-TNR nanoparticles and Au-TNR/PVDF nanocomposites. Meanwhile, no PVDF diffraction peaks were observed in the Au-TNR/PVDF nanocomposite sample, which can be attributed to the semicrystalline nature of PVDF, which is shielded by the stronger crystalline diffraction intensity of the TNRs compared to PVDF.

**Figure 4:**
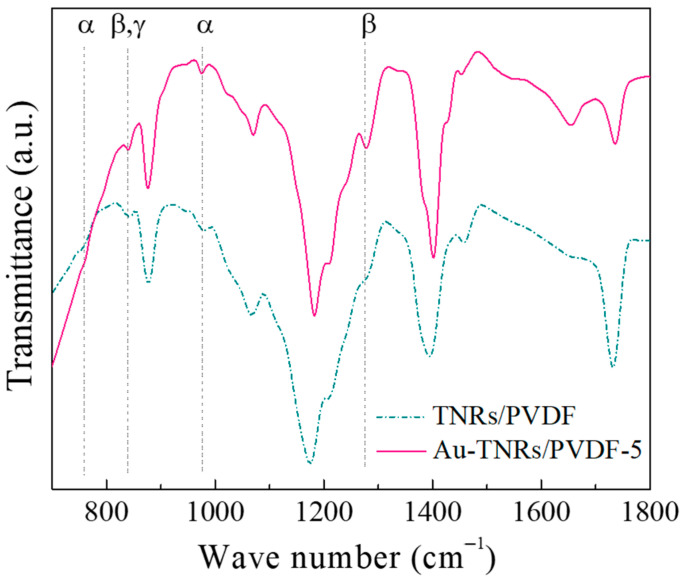
FTIR spectra of the TNR/PVDF and Au-TNR/PVDF-5 nanocomposites.

The FTIR spectra of the PVDF polymer nanocomposites filled with the TNRs and Au-TNRs are shown in Figure 4. Both nanocomposite systems consisted of α-, β-, and γ-PVDF phases. Weak transmittance bands observed at 766 and 976 cm^−1^ are attributed to the nonpolar α-phase [4], consistent with the XRD result (Figure 3). As the characteristic bands of the β- and γ-phase overlapped at 840 cm^−1^, they were difficult to distinguish. However, the characteristic band at 1279 cm^−1^ only corresponds to the β-phase [4]. As shown in Figure 4, the transmittance intensity of the β-phase for the three-phase Au-TNR/PVDF-5 composite is stronger than that of the two-phase TNR/PVDF composite, particularly at 1279 cm^−1^. To estimate the %β-phases in the nanocomposites, the absorption ratios of the β- and α-phase were compared. Equation (3) was used to quantify the relative fraction of the β-phase (F(β)) [4], assuming that only the β- and α-phase exist:

(3) $$F\left(\beta \right)=\frac{A_{\beta }}{\left(K_{\beta }/K_{\alpha }\right)A_{\alpha }+A_{\beta }}$$

where A_α_ and A_β_ are the absorption bands at 766 and 840 cm^−1^, respectively, and K_α_ and K_β_ are the absorption coefficients of the respective bands (K_α_ = 6.1 × 10^4^ and K_β_ = 7.7 × 10^4^ cm^2^·mol^−1^). The calculated F(β) of the two-phase and three-phase nanocomposites were 0.220 and 0.331, respectively. The negative charge of the Au nanoparticles causes an increase in amount of the polar β-phase of the PVDF nanocomposites [44], leading to a Au-TNR/PVDF nanocomposite with a significantly enhanced ε′ [45].

**Figure 5:**
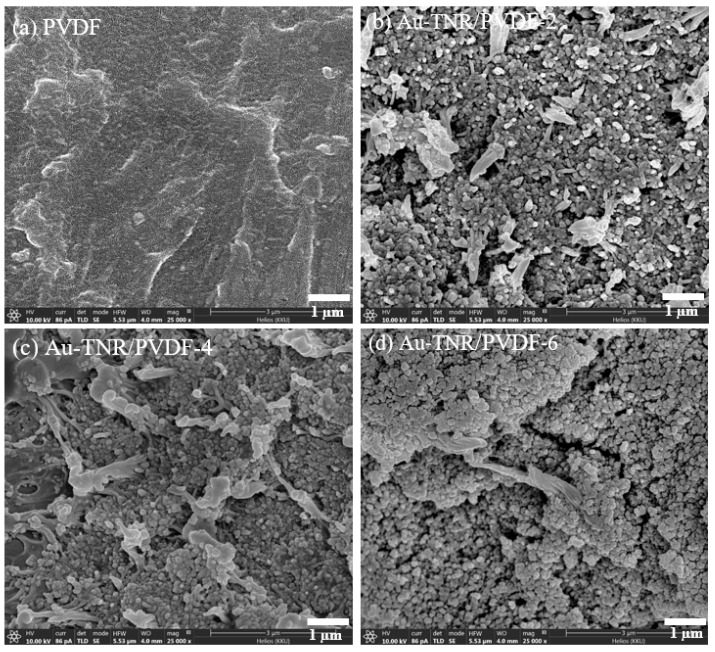
SEM cross-section images of (**a**) PVDF, (**b**) Au-TNR/PVDF-2, (**c**) Au-TNR/PVDF-4, and (**d**) Au-TNR/PVDF-6.

The fracture cross-sectional images of the nanocomposites containing various Au-TNR hybrid particles are shown in Figure 5. The microstructure of the PVDF polymer is shown in Figure 5a and reveals that the PVDF molecules form a continuous phase. Figure 5b,c show the microstructures of the Au-TNRs/PVDF-2 and Au-TNRs/PVDF-4 nanocomposites. The Au-TNR hybrid nanoparticles are dispersed homogeneously in the PVDF matrix without aggregation. Some air voids and Au-TNR nanoparticle aggregation were observed with increasing Au-TNR hybrid particle content, as exemplified by Au-TNR/PVDF-6, as shown in Figure 5d.

**Figure 6:**
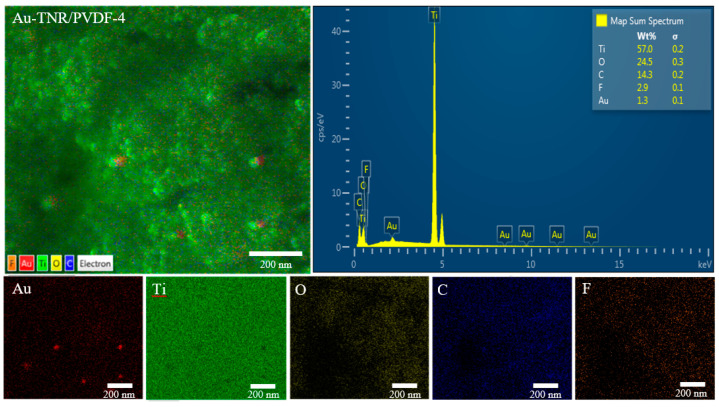
Element mapping and EDS‒FESEM characterization of Au-TNR/PVDF-4.

SEM element maps and EDS were employed to further confirm the existence of Au in the three-phase nanocomposites. As shown in Figure 6, the microstructure of Au-TNR/PVDF-4 exhibited Au clusters dispersed on the TNR surfaces that are surrounded by the PVDF matrix. EDS was used to determine that Au, Ti, O, C, and F are present in the nanocomposite at levels of 1.3, 57, 24.5, 14.3, and 2.9 wt%, respectively.

**Figure 7:**
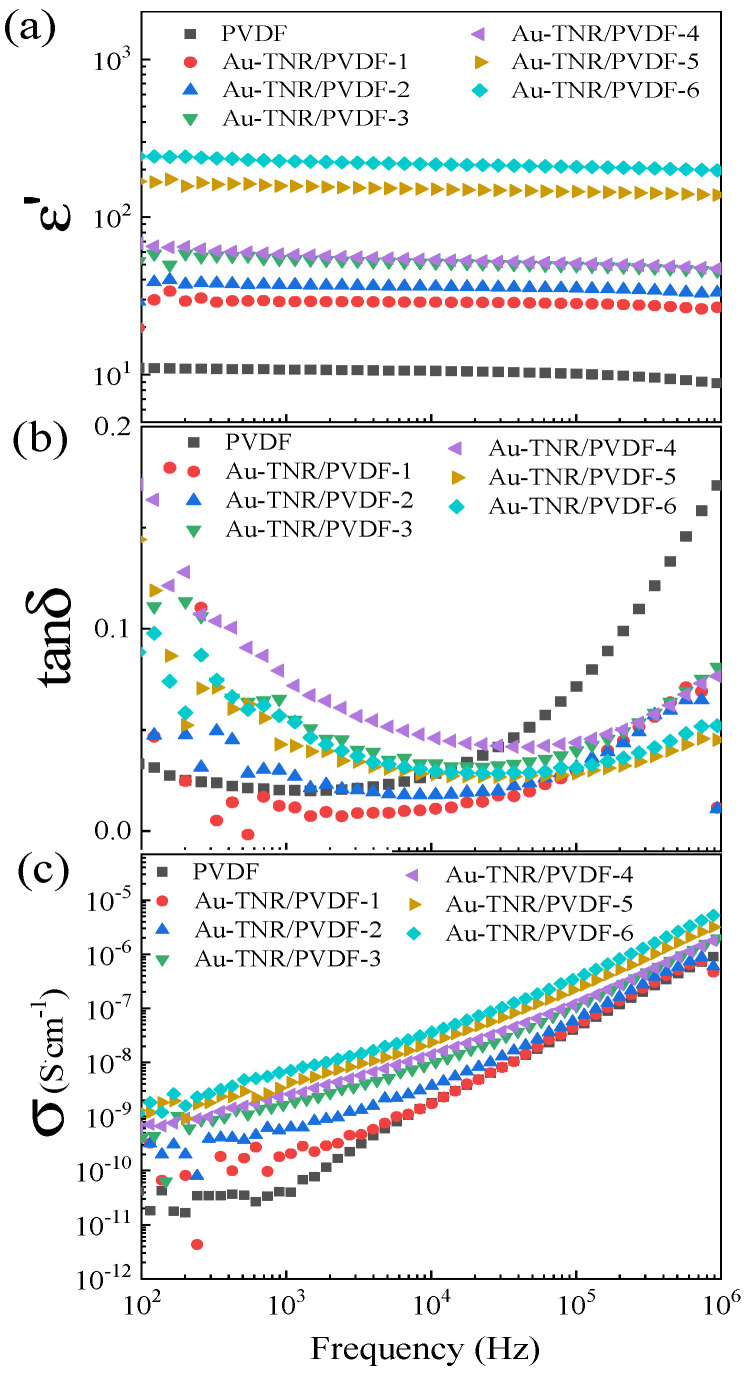
Frequency dependence of (**a**) ε′, (**b**) tanδ, and (**c**) σ for nanocomposites with varying amounts of Au-TNRs.

The frequency dependences of ε′, tanδ, and σ_ac_ of nanocomposites with different volume fractions of Au-TNRs (*f*_Au-TNRs_) at room temperature are shown in Figure 7. As shown in Figure 7a, the ε′ increased with increasing *f*_Au-TNRs_. A significant enhancement in ε′ was achieved by incorporating small amounts of Au and TNR nanoparticles in the nanocomposite. The enhanced ε′ value of the Au-TNR/PVDF-6 composite was ~226 at 1 kHz, which is ~20 times larger than that of a pure PVDF polymer (ε′ ≈ 10.78). The increase in ε′ for the three-phase Au-TNR/PVDF nanocomposites can be ascribed to the formation of Au-TNR hybrid nanoparticles. A large amount of blocked charges at the interface between TNR-PVDF and Au-PVDF can enhance interfacial polarization, which is known as Maxwell–Wagner–Sillars (MWS) polarization [6,46]. Therefore, in an electric field, the enhanced interfacial polarization enhances the ε′ of the Au-TNR/PVDF nanocomposites. Another factor is the semiconductor nature of the TNR nanoparticles, which can produce interfacial polarization over a wide range of frequencies. Moreover, the ε′ behavior of each sample exhibits a similar trend in the 10^2^–10^6^ Hz range. Meanwhile, the tanδ values of the Au-TNR/PVDF nanocomposites decreased as the frequency was increased to approximately 10^4^ kHz and gradually increased at higher frequencies, as shown in Figure 7b. This increase in tanδ is generally consistent with the dielectric relaxation of the pure PVDF polymer [6]. Considering a low-frequency range, tanδ of the Au-TNR/PVDF nanocomposites increased with increasing *f*_Au-TNRs_. The increased tanδ value as a result of increased filler loading is attributed to the conduction of free charge carriers [6,47], which corresponds to the increase in *f*_Au-TNRs_. Furthermore, for the composites with high filler loading, it is observed that tanδ continuously increases with decreasing frequency from 10^3^ to 10^2^ Hz. This observation was resulted from the conduction of free charge carriers, which is more prominent in a low-frequency range. The increase in tanδ in the high-frequency range is attributed to the α_a_ relaxation from the glass transition in the PVDF polymer [6,48]. Th tanδ of the nanocomposite increases slowly with increasing Au-TNR content. Interestingly, tanδ is exceptionally low for all nanocomposites at 1 kHz. The maximum value of tanδ is less than 0.08 at a frequency of 1 kHz. The tanδ value of Au-TNR/PVDF-6 is 0.05, which is much lower than values obtained in other work (tanδ > 0.1) that used Ag@TiO_2_ as fillers [34,35,37,49]. As shown in Figure 7c, the σ_ac_ value of the Au-TNR/PVDF nanocomposite increased slightly with increasing Au-TNR content. At *f*_Au-TNRs_ = 0.624, the σ_ac_ value of the nanocomposite was only 6.58 × 10^−9^ S·cm^−1^ at 1 kHz, which is lower than that of the other three-phase composite systems (>10^−7^ S·cm^−1^) [34,35]. These results confirm that no conducting network is formed, indicating that the Au-TNR-PVDF nanocomposites exhibit good insulation properties.

**Figure 8:**
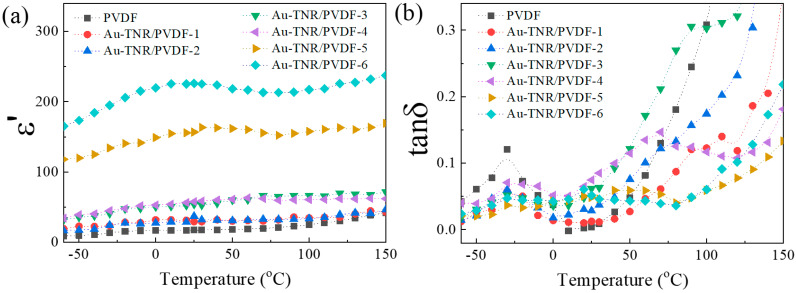
Temperature dependence of (**a**) ε′ and (**b**) tanδ for nanocomposites with varying amounts of Au-TNRs.

Figure 8 shows the ε′ and tanδ of Au-TNR/PVDF at 1 kHz as functions of temperature. As shown in Figure 8a, steady values of ε′ were observed for almost all nanocomposites with increasing temperature. Only Au-TNR/PVDF-5 and Au-TNR/PVDF-6 exhibited ε′ values that were slightly temperature dependent. Figure 8b shows the tanδ relaxation peaks in the pure PVDF polymer. The first relaxation was observed between −40 and 0 °C, which can be attributed to the β-relaxation of PVDF. The second relaxation was observed at a temperature above 40 °C, which can be attributed to the α-relaxation [50].

**Figure 9:**
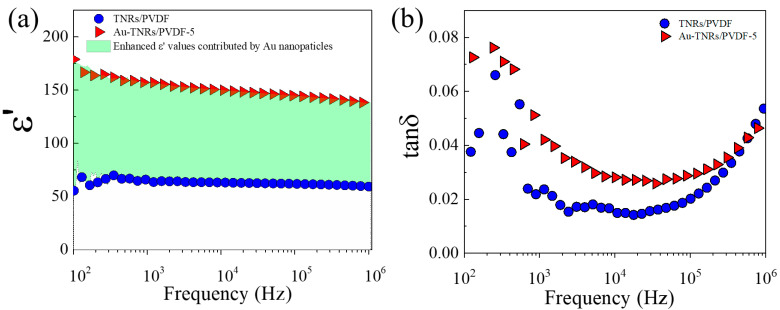
Frequency dependence of (**a**) ε′ and (**b**) tanδ for TNR/PVDF and Au-TNR/PVDF-5 at 20 °C; the different ε′ values (green area) resulted from the Au nanoparticles.

Figure 9a shows the ε′ values of TNR/PVDF and Au-TNR/PVDF-5 as a function of frequency. The ε′ value of the three-phase nanocomposite (Au-TNR/PVDF-5) was found to be much higher than that of the two-phase nanocomposite (TNR/PVDF) (with nearly the same total volume fraction of filler) in the 10^2^–10^6^ Hz frequency range, which indicates that the addition of a small amount of Au nanoparticles can result in a significant enhancement in the ε′ of a polymer composite. Interestingly, the tanδ value of the Au-TNR/PVDF-5 nanocomposite at 1 kHz was 0.048. These excellent dielectric properties of Au-TNR/PVDF are not only due to the introduction of the Au-TNR hybrid nanoparticles, but also due to the increasing polar β-phase in the PVDF matrix, which was confirmed by FTIR spectroscopy (Figure 4). The large interfacial area of the semiconducting TNRs is one of the most important factors that significantly increases the dielectric response in the nanocomposite. As shown in Figure 9b, although tanδ of the Au-TNR/PVDF-5 nanocomposite was increased over the measured frequency range compared to that of the two-phase TNR/PVDF nanocomposite, the obtained tanδ value was lower than 0.08 in the frequency range of 10^2^–10^6^ Hz.

**Figure 10:**
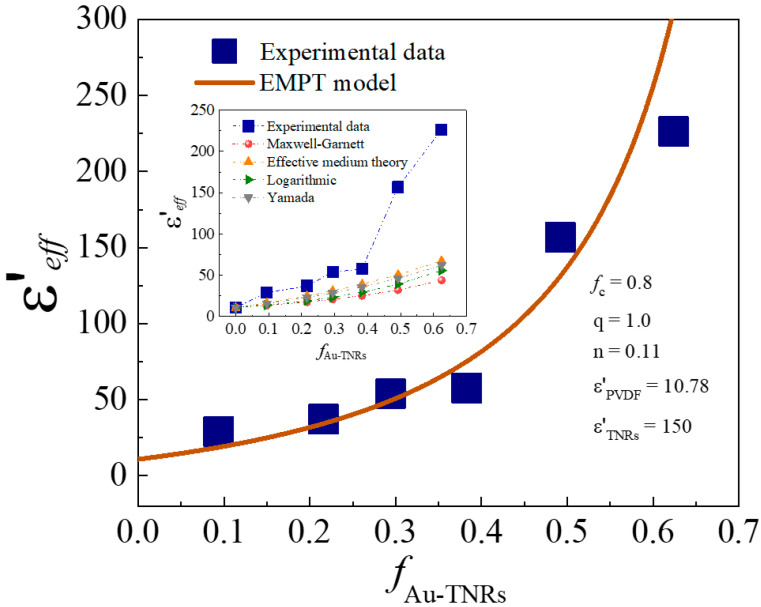
Experimental data of ε′ for the Au-TNR/PVDF nanocomposites at 1 kHz and 20 °C fitted by the effective medium theory (EMPT) model; inset is the experimental data of ε′ for the Au-TNR/PVDF nanocomposites fitted by two-phase various theoretical models.

The ε′ values of the Au-TNR/PVDF nanocomposites could not be fitted to two-phase composite models consisting of a ceramic and a polymer (e.g., effective medium theory (EMT), Maxwell–Garnett, Yamada, logarithmic [5,51]) with high Au-TNR contents, as demonstrated in the inset of Figure 10. This is due to interfacial polarization at the interface between fillers and PVDF polymer matrix. Moreover, the ε′ values of the Au-TNR/PVDF nanocomposites could not be fitted to the percolation model, which is employed for metal/polymer dual phases. As shown in Figure 10, the dielectric behavior of the Au-TNR/PVDF nanocomposites is in good agreement with the EMPT model [35,52], which combines the EMT model with percolation theory, as shown in Equation (4):

(4) $$\varepsilon_{\mathrm{eff}}=\varepsilon_{\mathrm{PVDF}}\left[1+\frac{f_{\mathrm{TNRs}}\left(\varepsilon_{\mathrm{TNRs}}-\varepsilon_{\mathrm{PVDF}}\right)}{\varepsilon_{\mathrm{PVDF}}+n\left(1-f_{\mathrm{TNRs}}\right)\left(\varepsilon_{\mathrm{TNRs}}-\varepsilon_{\mathrm{PVDF}}\right)}\right]{\left|\frac{f_{c}-f}{f}\right|}^{-q}$$

where ε_eff_ is the effective ε′ of the Au-TNR/PVDF composite, *f*_TNRs_ is the volume fraction of the TNRs, *f*_c_ is the percolation threshold, ε_PVDF_ is the ε′ of PVDF (ε_PVDF_ = 10.78), ε_TNRs_ is the ε′ of TNRs (ε_TNRs_ = 150), n is the morphology fitting factor, and *q* is the critical exponent. Due to the semiconducting nature of TNRs and conducting nature of Au nanoparticles, *f* is assigned as the volume fraction of Au-TNR hybrid particles, which can influence the percolation behavior of the composites. For the curve fitted using the EMPT model, the optimum fitting parameters were determined to be: n = 0.11, *q* = 1.0, and *f*_c_ = 0.8. It is worth noting that n and *q* are very close to those reported for the Ag-BaTiO_3_/PVDF (n = 0.11) [52] and the Ni-BaTiO_3_/PVDF (*q* = 1.0) [23], respectively. The percolation threshold is expected to occur at a high filler loading (*f*_c_ = 0.8), which is much higher than the maximum filler loading used in this current study, and is due to the small amount of conductive Au nanoparticles used and the hybrid structure of the Au-TNR particles. Therefore, the percolation network (or conduction pathway) would not be formed in the Au-TNR/PVDF composite because the hybrid structures of the Au-TNRs prevent the formation of conducting pathways because the randomly grown Au nanoparticles do not continuously coat the TNR surface. The large increase in the ε′ value is primarily attributed to interfacial polarization between the Au–PVDF, Au–TNR, and TNR–PVDF interfaces.

## 4. Conclusions

This study presented a novel method for successfully achieving high ε′ and low tanδ in three-phase PVDF polymer-matrix nanocomposites. The dielectric properties of a PVDF polymer improved significantly by incorporating conductive Au nanoparticles and semiconductive TNRs with enlarged interfacial areas. The Au nanoparticles were discretely attached to the TNR surfaces to enhance interfacial polarization and simultaneously prevent the formation of conducting pathways in the insulative PVDF matrix. As a result, a high ε′ (~157) and low tanδ (~0.05) were obtained in the three-phase nanocomposite filled with 1.8 vol% Au and 47.4 vol% TNRs. The dielectric response in the two-phase TNR/PVDF composite increased by more than a factor of two after introducing small amounts of Au nanoparticles. This dielectric behavior is described using the EMPT model. The results indicate that Au nanoparticles significantly contribute to enhancing interfacial polarization and creating a more polar β-PVDF phase, which increases ε′. In contrast, due to the small amount of Au nanoparticles used and their discrete growth on the TNRs, the value of tanδ remained low. To further investigate the possible use of the Au-TNR/PVDF nanocomposites in capacitor applications, fabrication conditions that produce nanocomposite thin films need be studied.

## Figures and Tables

**Table 1:** Volume fraction of Au (*f*_Au_), TNRs (*f*_TNRs_), Au-TNRs (*f*_Au-TNRs_), ε′, tanδ, and σ_ac_ at 1 kHz and room temperature for nanocomposites with varying filler amounts.

Sample	*f*_Au_	*f*_TNRs_	*f*_Au‒TNRs_	ε′	tanδ	σ_ac_ (10^−11^ S·cm^−1^)
PVDF	0	0	0	10.8	0.020	4.1
Au-TNR/PVDF-1	0.005	0.089	0.094	29.1	0.012	20.3
Au-TNR/PVDF-2	0.010	0.206	0.216	37.1	0.028	59.1
Au-TNR/PVDF-3	0.013	0.281	0.294	53.8	0.062	188.6
Au-TNR/PVDF-4	0.016	0.367	0.383	57.7	0.075	242.8
Au-TNR/PVDF-5	0.018	0.474	0.492	156.7	0.048	427.7
Au-TNR/PVDF-6	0.021	0.603	0.624	226.3	0.052	657.6
TNR/PVDF	0	0.5	0	65.9	0.028	103.5

## Data Availability

The data presented in this study are available in article.
